# Combined *FCGR2A* (131H/R) and *FCGR3A* (158F/V) genotypes and their gender-specific association with chronic and refractory immune thrombocytopenia in Palestinian children

**DOI:** 10.3389/fmed.2025.1606953

**Published:** 2025-10-14

**Authors:** Khitam Amer, Johnny Amer, Adham Abu Taha, Wisam Baker, Awad Abuhamed, Ahmad Salhab

**Affiliations:** ^1^Department of Allied and Applied Medical Sciences, An-Najah National University, Nablus, Palestine; ^2^Martyr Dr. Khalil Suleiman Governmental Hospital, Jenin, Palestine; ^3^Department of Pediatrics, Rafidia Government Surgical Hospital, Nablus, Palestine

**Keywords:** immune thrombocytopenia, Fc gamma receptors (FcγRs), *FCGR2A* (131H/R), *FCGR3A* (158F/V), polymorphisms

## Abstract

**Background:**

Immune thrombocytopenia (ITP) is a common pediatric autoimmune disorder characterized by low platelet counts and heightened bleeding risk. Fc gamma receptors (FcγRs), particularly *FCGR2A* (131H/R) and *FCGR3A* (158F/V), mediate immune responses and may influence ITP susceptibility and progression. Gender-related genetic variation has been proposed but remains underexplored, particularly in Middle Eastern pediatric populations. This study aimed to perform an exploratory assessment of the prevalence and potential clinical relevance of *FCGR2A* and *FCGR3A* polymorphisms, including gender-based tendencies, in Palestinian children with ITP.

**Methods:**

A multicenter case-control study included 40 proven pediatric ITP patients (20 males, 20 females; mean age 6.76 ± 4.13 years) and 80 age- and sex-matched healthy controls. Genotyping was performed using PCR-RFLP and nested PCR. Genotype frequencies were correlated with disease phenotype and sex.

**Results:**

No statistically significant differences in genotype distributions were observed between ITP cases and controls for either *FCGR2A* (HH: 17.5%, HR: 62.5%, RR: 20.0%) or *FCGR3A* (FF: 25.0%, FV: 55.0%, VV: 20.0%) (*p* > 0.05). However, a secondary, exploratory analysis for gender-specific trends yielded noteworthy observations: *FCGR2A*-HH was numerically more frequent in male ITP patients (57.4%) than in females (42.8%), while HR was lower in males (48% vs. 52%). Similarly, *FCGR3A*-VV occurred in 62.5% of male ITP patients versus 37.5% in females. Furthermore, the combined *HR/FV* genotype (32.5%) showed a non-significant trend of association with chronic ITP (69.2%), while the *VV/HH* genotype, although rare (5%), was linked to 50% of refractory presentations.

**Conclusion:**

This exploratory study found no statistically significant association between *FCGR2A* and *FCGR3A* polymorphisms and overall ITP susceptibility in the full cohort. However, the observed trends, particularly the distinct gender-based distribution of specific genotypes and the association of combined genotypes with chronic and refractory disease, suggest that these genetic markers may play a role in disease progression. Further investigation in a larger, appropriately powered study is warranted to validate these findings and to understand their potential to guide personalized treatment approaches for pediatric ITP.

## Introduction

Immune thrombocytopenia (ITP) is an autoimmune hematologic disorder characterized by a markedly reduced platelet count (< 100 × 10^9^/L) that predisposes affected children to a spectrum of bleeding manifestations from mild mucocutaneous hemorrhages to severe, life-threatening events (1). As the most common cause of isolated thrombocytopenia in the pediatric population, ITP represents a significant clinical challenge, with an incidence that underscores its public health relevance (2). Clinically, ITP is categorized into acute, persistent, and chronic forms; while many acute cases resolve spontaneously, approximately 20%–30% of children experience a progression to chronic ITP, necessitating long-term management and monitoring (3). The pathogenesis of ITP is complex and multifactorial, involving both humoral and cellular immune dysregulation that culminates in the premature destruction and impaired production of platelets. In recent years, there has been an increasing focus on the role of genetic factors in modulating both the susceptibility to and the clinical course of ITP. Fc gamma receptors (FcγRs) on platelets, particularly *FCGR3A* and *FCGR2A*, are integral in modulating the immune response and platelet function in chronic ITP. These receptors play a crucial role in platelet destruction, as they interact with autoantibodies targeting platelets. Genetic polymorphisms in these receptors, such as *FCGR3A* 158F/V and *FCGR2A* 131H/R, can significantly impact platelet response, disease progression, and the severity of chronic ITP (4). The *FCGR2A*-131H allele is known to confer broader IgG subclass binding capabilities compared to the 131R variant, potentially enhancing the phagocytosis of antibody-coated platelets. In parallel, the *FCGR3A*-158V allele exhibits a higher affinity for IgG1 and IgG3, the predominant subclasses involved in the autoimmune process of ITP (5). Emerging evidence also suggests that these functional differences may influence treatment responses, particularly to therapies such as intravenous immunoglobulin (IVIg), which remains a cornerstone in ITP management (6, 7). Despite significant advancements in our understanding of the immunogenetics underlying ITP, there remains a paucity of data on the prevalence and clinical impact of these FCGR polymorphisms in specific populations especially among children in the Arab world (8). Given the potential variability in genetic backgrounds and environmental influences, investigating these polymorphisms in diverse cohorts is critical for elucidating their role in disease susceptibility and progression. Therefore, this study aims to determine the prevalence of the *FCGR2A* (131H/R) and *FCGR3A* (158F/V) gene polymorphisms in children with primary ITP admitted to four major hospitals in the West Bank/Palestine between 2019 and 2023. By correlating the genetic profiles with clinical phenotypes, this research seeks to ascertain whether these variants serve as reliable biomarkers for disease susceptibility, severity, and chronicity.

## Materials and methods

### Study design and patients criteria

This multicenter and retrospective case-control study was performed following IRB approval from An-Najah National University/Palestine (Mas. Nov. 2023/30) and was approved by the Palestinian Ministry of Health/Education in Health and Scientific Research Unit. In the absence of any official data on the prevalence of ITP in Palestine, between February and May of 2024, we retrieved medical data from the hospital information system on documented cases of children with ITP who were referred to pediatric units at four different hospitals: the Martyr Dr. Khalil Suleiman Governmental Hospital in Jenin, the Palestine Medical Complex in Ramallah, Rafidia Governmental Surgical Hospital and An-Najah National University Hospital, both located in Nablus, between 2019 and 2023. Control samples were kindly obtained from Medicare labs in Palestine and from the Pediatric Unit of Rafidia Government Surgical Hospital in Nablus city. ITP cases were diagnosed based on appropriate history taking, clinical presentation, and laboratory findings to rule out other possible causes of thrombocytopenia. There were 105 registered cases (49 females and 56 males): 53 acute, 39 chronic, six persistent, and seven refractories. All demographic and clinical information regarding them was retrieved from the Hospitals’ information system. Patients with secondary immune thrombocytopenia, younger than one year and older than eighteen, who refused to sign informed consent were excluded.

### Blood sampling and DNA extraction

Ethylenediaminetetraacetic acid (EDTA) blood samples (3–5 ml) were obtained from each participant, and then the genomic DNA extraction was performed using the Gene JET™ Whole Blood Genomic DNA purification Mini Kit (Thermo Scientific, catalog number K0782). The extracted DNA was stored at −20 °C until use.

### *FCGR2A* 131H/R gene genotyping using PCR and PCR-RFLP analysis

**FIGURE 1:**
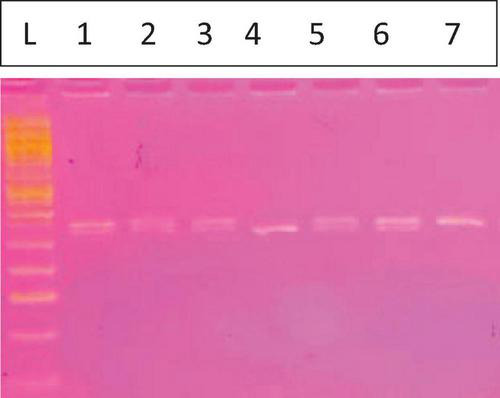
Fc gamma receptor IIa131H/R. The L is the 50 bp ladder, Lanes 1, 2, 3, 5, and 6 for HR (337, 316 bp) lane 4 showing 1 band for the RR mutant type (316 bp) and lane 7 for the HH wild type (337 bp).

The FcγRIIa-131R/H genotyping was performed using the PCR-restriction fragment length polymorphism (PCR-RFLP) method. The primers for the *FCGR2A* gene were designed according to the protocol described by (9), as specified in Table 1. After that, the amplified PCR product was digested at 60 °C for 2 h using 20U of the *Bst*UI restriction endonuclease (New England Biolabs). Digested products were electrophoresed on a 3.5% agarose gel. The R alleles generated two 316 and 21 bp fragments, while the FCGR2A H allele was visualized as a 337 bp fragment (Figure 1) as described by Cartron et al. (9).

**TABLE 1:** Primers used for genotyping *FCGR3A* and *FCGR2A*II.

Genotyping of *FCGR3A*158 V/F gene	Primer sequence	Amplicon size bp
Forward	5′-ATATTTACAGAATGGCACAGG-3′	1.2 Kb
Reverse	5′-GACTTGGTACCCAGGTT GAA-3′	
Forward	5′-ATCAGATTCGATCCTACTTCTGCAGGGGGCAT-3′	94 bp
Reverse	5′-ACGTGCTGAGCTTGAGTGATGGTGATGTTCAC-3′	
**Genotyping of *FCGR2A* 131H/R gene**	**Primer sequence**	
Forward	5′-GGAAAATCCCAGAAATTCTCGC-3′	337 bp
Reverse	5′-CAACAGCCTGACTACCTATTACGCGGG-3′	

### *FCGR3A* 158V/F gene genotyping using an allele-specific restriction analysis based on nested PCR

**FIGURE 2:**
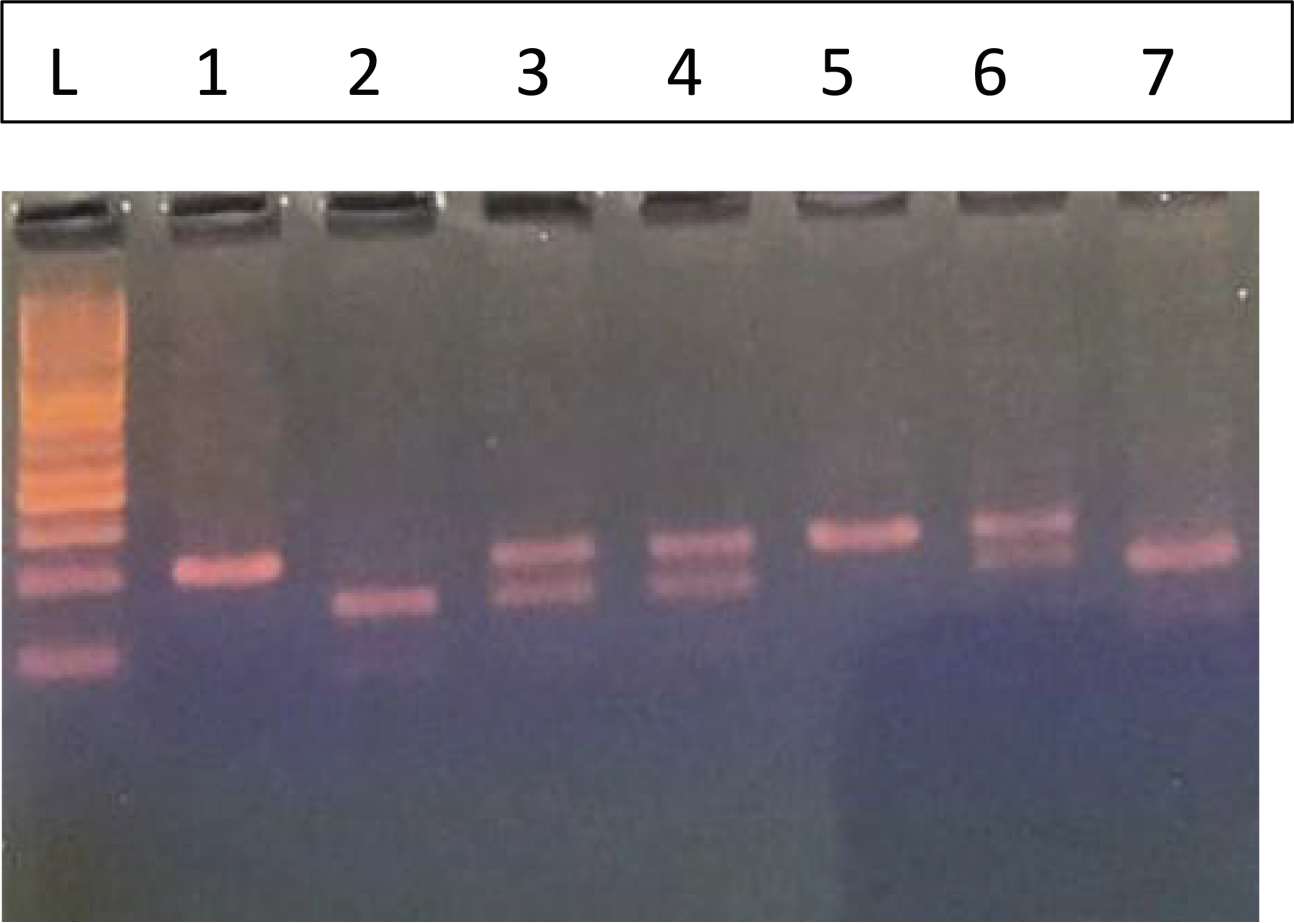
Fc gamma receptor IIIa158F/V. L. 50 bp Ladder, The FF (Wild) phenotype is represented by lanes 1 and 5 with one band 94 bp, the VV (Homozygous) by lanes 2 and 7 (61, 33 bp), and the FV (Heterozygous) by lanes 3, 4, and 6 (94, 63, and 33 bp).

*FCGR3A*–158V/F genotyping was performed as previously described by Cartron et al. using nested PCR followed by allele-specific restriction enzyme digestion. The initial PCR assay to amplify a 1.2 kb fragment. Then, a second PCR reaction was done to amplify part (94 bp) of the amplified DNA produced by the first reaction using primers shown in Table 1. Then, the amplified DNA was digested with 10U *NLaIII* restriction endonuclease (Thermo-Scientific) at 37 °C for 2 h. DNA fragments were visualized on 3.5% gel; the V allele generated two pieces of 61 and 33 bp, while the CFGR3A F allele manifested as a 94 bp fragment (Figure 2) (9).

For both polymorphisms, random samples were selected and tested in duplicate to ensure the result. The results were read by two lab technicians, and a positive or mutant sample was included in each run.

### Statistical analysis

The Statistical Package for Social Science (IBM SPSS statistics 21 for Windows) was used to analyze the data. The range, mean, standard deviation, and median are how numerical data are presented. The chi-square test determined the genotype distribution between patients and controls. The odds ratio (OR) and 95% confidence interval (CI) were computed to estimate risk. A probability value (*P*-value) less than 0.05 is considered statistically significant.

## Results

### FcγRIIa H131R genotyping by PCR-RFLP

Genotyping of the FcγRIIa H131R polymorphism (Figure 1) was performed on the collected samples using the PCR-RFLP method. Figure 1 displays a representative image of the agarose gel electrophoresis results. Lane L contains a 50 bp DNA ladder, which was used as a molecular weight marker to confirm the size of the digested fragments. Subsequent lanes illustrate the three possible genotypes. The FcγRIIa H allele was identified by a 337 bp fragment, while the R allele was identified by a 316 bp fragment following digestion with the *Bst*UI restriction enzyme. The smaller 21 bp fragment resulting from the R allele digestion was not visible on the 3.5% agarose gel. The following distinct banding patterns corresponded to the different genotypes:

1. Heterozygous (HR): Lanes 1, 2, 3, 5, and 6 show two bands corresponding to the sizes of 337 and 316 bp. This pattern is characteristic of the heterozygous genotype, indicating the presence of both the H and R alleles.
2. Homozygous Mutant (RR): Lane 4 exhibits a single band at 316 bp. This pattern indicates a homozygous mutant genotype where both alleles are the R variant.
3. Homozygous Wild-type (HH): Lane 7 displays a single band at 337 bp. This pattern is consistent with the homozygous wild-type genotype, indicating the presence of two H alleles.

The distribution of these genotypes was determined for the entire sample cohort, revealing a clear visual representation of each genotypic class.

### FcγRIIIa V158F genotyping by PCR-RFLP

Genotyping for the FcγRIIIa V158F polymorphism was conducted using the PCR-RFLP method, and the results of the gel electrophoresis are presented in Figure 2.

The leftmost lane (L) contains a 50 bp DNA ladder, which was used to determine the sizes of the DNA fragments. The analysis revealed three distinct banding patterns, corresponding to the FF, VV, and FV genotypes.

1. Homozygous Wild-type (FF): As shown in lanes 1 and 5, this genotype is characterized by a single band at 94 bp. This fragment represents the undigested PCR product from the F allele, which lacks the restriction enzyme recognition site.
2. Homozygous Mutant (VV): Lanes 2 and 7 show a two-band pattern with fragments at 61 bp and 33 bp. This result is consistent with the homozygous mutant genotype. The V allele contains the restriction site, which leads to the digestion of the PCR product into these two fragments.
3. Heterozygous (FV): The heterozygous genotype is represented in lanes 3, 4, and 6. These lanes display a three-band pattern, with fragments at 94, 61, and 33 bp. This pattern indicates the presence of both the F allele (uncut, 94 bp) and the V allele (cut, 61 and 33 bp).

These distinct banding patterns enabled the successful classification of each sample into its corresponding FcγRIIIa V158F genotype.

### Demographic and clinical profile of immune thrombocytopenic purpura

To understand the characteristics of immune thrombocytopenic purpura (ITP) cases within our clinical setting, it was essential to initially examine the broader patient demographics and clinical profiles. Table 2 summarizes the demographic and clinical characteristics of the registered ITP cases (*N* = 105) and details the subset of participants (*N* = 40) included in the study between 2019 and 2023. Overall, among the 105 registered ITP cases, there were 56 males (53.3%) and 49 females (46.7%). In terms of disease duration, 53 cases (50.5%) were classified as acute, 39 cases (37.1%) as chronic, six cases (5.7%) as persistent, and seven cases (6.7%) as refractory. The registered cases were distributed as follows: Rafidia Hospital (33 cases, 31.4%), followed by NNUH (21 cases, 20.0%), PMC (17 cases, 16.2%), NNUH/Rafidia (16 cases, 15.2%), Jenin (15 cases, 14.3%), and NNUH/Jenin (three cases, 2.9%). Of these, 40 participants were selected for further analysis. Within the male group, 20 out of 56 cases (35.7%) were included, while 20 out of 49 female cases (40.8%) were selected. To this end, 120 participants were enrolled, including 40 ITP patients (representing 38% of the 105 registered cases) and 80 age- and sex-matched healthy controls. The ITP group, with a mean age of 6.76 ± 4.13 years, consisted of an equal number of males (20; 50%) and females (20; 50%), while the control group included 44 males (55%) and 36 females (45%). Among the ITP patients, the disease was categorized as acute in 45% of cases, chronic in 42.5%, persistent in 7.5%, and refractory in 5% (Table 3). Statistical analysis revealed no significant difference in gender distribution between the patient and control groups (*p* = 0.605), thereby confirming that the demographic characteristics of the two groups were comparable. In conclusion, the balanced distribution of demographic variables and the detailed categorization of ITP duration in the patient group provide a robust foundation for the subsequent analysis of *FCGR2A* and *FCGR3A* polymorphisms. This representativeness minimizes potential confounding factors and strengthens the validity of the genetic association analyses conducted in this study.

**TABLE 2:** The demographic and clinical characteristics of 105 registered immune thrombocytopenia (ITP) cases.

Patients (*n* = 105)	Gender		Duration			
	Male	Female	Acute	Chronic	Persistent	Refractory
Registered cases (105)	56 (53.3%)	49 (46.7%)	53 (50.5%)	39 (37.1%)	6 (5.7%)	7 (6.7%)
Hospital name	Rafidia	Jenin	NNUH	NNUH/Rafidia	NNUH/Jenin	PMC
	33 (31.4%)	15 (14.3%)	21 (20%)	16 (15.2%)	3 (2.9%)	17 (16.2%)

**TABLE 3:** General participant’s characteristics and the course of the disease.

Variable		Total	ITP		Control group		*P*-value
		*N* (%)	*N*	%			
Gender	Male	64 (53.3)	20	50.0%	44	55.0%	0.605
	Female	56 (46.7)	20	50.0%	36	45.0%	–
Duration (ITP: *n* = 40)	Acute	–	18	45.0%	–	–	–
	Chronic	–	17	42.5%	–	–	–
	Persistent	–	3	7.5%	–	–	–
	Refractory	–	2	5.0%	–	–	–

### Association between *FCGR2A* and *FCGR3A* polymorphisms and clinical course of ITP

Investigating *FCGR2A* and *FCGR3A* polymorphisms in this cohort were to determine whether variations in these IgG-binding receptors could influence ITP susceptibility and clinical presentation. Table 4 compares the genotype and allele frequencies of both polymorphisms between 40 ITP patients and 80 healthy controls. For FCGR2A, the frequencies of the HH, HR, and RR genotypes in the ITP patient group were 17.5%, 62.5%, and 20.0%, respectively, whereas in the control group, these genotypes were 25.0%, 52.5%, and 22.5%. No statistically significant difference emerged (*p* = 0.543). A notable observation was that the heterozygous HR genotype occurred in approximately 52% of female patients and in 52% of male controls. Allele frequencies (H and R) also showed no significant difference between groups (*p* = 0.82). Similarly, for *FCGR3A*, the ITP patients exhibited FF, FV, and VV genotypes at 25.0%, 55.0%, and 20.0%, respectively, compared with 23.8%, 53.8%, and 22.5% in controls (*p* = 0.950). Although a higher frequency of the FV phenotype was noted in female patients (59.09%) compared with male controls (60.46%), this did not reach statistical significance. Allele frequencies (F and V) were comparable between the two groups (*p* = 0.88). In conclusion, neither FCGR2A nor FCGR3A polymorphisms showed a statistically significant association with ITP in this cohort. These findings suggest that, at least in this population, variations in these Fc gamma receptors do not appear to play a major role in determining disease susceptibility or phenotype.

**TABLE 4:** Comparison of the genotypes and allele frequencies of the *FCGR3A* and *FCGR2A* polymorphisms in patients and control groups.

Variable	Alleles		Total		Patient group (ITP)		Control group		*P*-value
			*N*	%	*n*	%	*n*	%	
FCGR3A	TT	Wild (FF)	29	24.2	10	25.0%	19	23.8%	0.950
	GG	Homozygous (VV)	26	21.7	8	20.0%	18	22.5%	
	TG	Heterozygous (FV)	65	54.2	22	55.0%	43	53.8%	
	T	T (158F)	–	–	–	52.5%	56	50.63%	0.88
	G	G (158V)	–	–	–	47.5%	104	49.37%	
FCGR2A	AA	Wild (HH)	27	22.5	7	17.5%	20	25.0%	0.543
	GG	Homozygous (RR)	26	21.7	8	20.0%	18	22.5%	
	AG	Heterozygous (HR)	67	55.8	25	62.5%	42	52.5%	
	A	A (H)	–	–	–	48.75%	82	51.25%	0.82
	G	G (R)	–	–	–	51.25%	60	48.75%	

### Gender-associated genotypic trends in *FCGR2A* and *FCGR3A* polymorphisms among pediatric ITP patients

To determine if specific *FCGR2A*131H/R and *FCGR3A*158F/V genotype distributions are associated with increased susceptibility to ITP, particularly in relation to gender, potentially guiding personalized diagnostic approaches. Data represented in Table 5 demonstrated gender distribution analysis and revealed that the prevalence of *FCGR2A*131 HH genotype among male ITP patients was 57.4% as compared to 42.8% in their female counterparts in a ratio of 1.33:1 (M: F ratio). In parallel, the prevalence of HH genotype among male healthy control was 35% as compared to 65% in their female counterparts in a ratio of 0.53:1 (M: F ratio), indicating a higher frequency of HH allele variation of the *FCGR2A*131 among ITP male patients. The prevalence of *FCGR2A*131 HR genotype among male ITP patients was 48% as compared to 52% in their female counterparts in a ratio of 0.9:1 (M: F ratio). In parallel, the prevalence of HR genotype among male healthy control was 52.5% as compared to 40.47% in their female counterparts in a ratio of 1.29:1 (M: F ratio), indicating a lower frequency of HR allele variation of the *FCGR2A*131 among ITP male patients. The RR population shows an equal distribution between male and female ITP patients and controls, with both groups having 50% of each gender in this category in a ratio of 1:1 (M:F ratio).

**TABLE 5:** The distribution of *FCGR2A*131H/R and *FCGR3A*158F/V genotypes among patients with immune thrombocytopenia (ITP) and healthy controls.

Genotype	ITP [*n* = 40 (%)]	Control (*n* = 80%)
*FCGR2A*131H/R	20 male (50%)/20 female (50%)	44 male (55%)/36 female (45%)
Total HH population	(7/40) 17.5%	(20/80) 25%
Male HH population	(4/7) 57.41%	(7/20) 35%
Female HH population	(3/7) 42. 85%	(13/20) 65%
Total HR population	(25/40) 62.5%	(42/80) 52.5%
Male HR population	(12/25) 48%	(25/42) 52.5%
Female HR population	(13/25) 52%	(17/42) 40.47%
Total RR population	(8/40) 20%	(18/80) 22.5%
Male RR population	(4/8) 50%	(9/18) 50%
Female RR population	(4/8) 50%	(9/18) 50%
***FCGR3A* 158 F/V**	**Patient (%)**	**Control (%)**
Total FF population	(10/40) 25%	(19/80) 23.75%
Male FF population	(3/10) 30%	(7/19) 36.8%
Female FF population	(7/10) 70%	(12/19) 63.2%
Total FV population	(22/40)55%	(43/80) 53.75%
Male FV population	(9/22) 40.9%	(26/43) 60.46%
Female FV population	(13/22) 59.09%	(17/43) 39.53%
Total VV population	(8/40) 20%	(18/80) 22. 5
Male VV population	(5/8) 62.5%	(7/18) 38.9%
Female VV population	(3/8) 37.5%	(11/18) 61.1%

Moreover, analysis of the *FCGR3A*158F/V genotype revealed that the prevalence of FF genotype among ITP male was 30% as compared to 70% in their female counterparts in a ratio of 0.42:1 (M: F ratio). In parallel, the prevalence of FF genotype among male healthy control was 36.8% as compared to 63.2% in their female counterparts in a ratio of 0.58:1 (M: F ratio), indicating a similar frequency of FF allele variation of the *FCGR2A*131 among ITP male patients. On the other side, FV genotype among ITP male was 40.9% as compared to 59.09% in their female counterparts in a ratio of 0.69:1 (M: F ratio). In parallel, the prevalence of FV genotype among male healthy control was 60.46% as compared to 39.3% in their female counterparts in a ratio of 1.53:1 (M: F ratio), indicating a lower frequency of FV allele variation of the *FCGR3A*158F/V genotype among ITP male patients. Additionally, In ITP patients, the VV allele was more prevalent in males (62.5%) than in females (37.5%), representing a male-to-female ratio of 1.66:1. In parallel, the prevalence of VV genotype among male healthy control was 38.9% as compared to 61.1% in their female counterparts in a ratio of 0.63:1 (M: F ratio), indicating a lower frequency of VV allele variation of the *FCGR3A*158F/V genotype among healthy male patients.

In conclusion, the findings suggest that there are notable gender-specific differences in the distribution *of FCGR2A*131H/R and *FCGR3A*158F/V genotypes among ITP patients, with distinct variations observed between male and female subjects. Specifically, the higher frequency of the *FCGR2A*131HH genotype in male ITP patients compared to healthy male controls, along with a lower frequency of the HR genotype in male patients, indicates a potential association with increased susceptibility to ITP in males. Similarly, the *FCGR3A*158F/V genotype analysis shows a trend of lower frequencies of FV and VV genotypes in male ITP patients compared to their healthy counterparts, further supporting the possibility of gender-specific genetic predispositions to ITP. These findings suggest that gender may play a significant role in the distribution of specific genotypes in ITP, potentially aiding in the development of more personalized diagnostic and therapeutic approaches for the condition.

### Genetic polymorphisms of *FCGR3A* 158F/V and *FCGR2A* 131H/R: key players in chronic immune thrombocytopenia

Analysis of combined genotypes of *FCGR2A* and *FCGR3A* polymorphisms revealed variability in disease severity among the studied cases (Table 6). The genotype combination FV-HR was the most prevalent, representing 32.5% of cases, primarily associated with chronic presentations (69.2%), followed by acute (23.1%) and persistent (7.7%) cases, with no refractory occurrences. Conversely, combinations FV-RR and VV-RR exclusively correlated with acute cases (100%), though these genotypes had low absolute frequencies (10% and 2.5%, respectively). The VV-HH combination showed significant severity, with 50% refractory cases, indicating a potential association with treatment resistance despite its low prevalence (5%). FF-HR exhibited a diverse clinical profile, predominantly chronic (57.1%) but also associated with acute, refractory, and persistent presentations. No cases were recorded for the FF-HH genotype combination. These findings suggest that specific combinations of FCGR2A and FCGR3A polymorphisms are linked with distinct clinical outcomes, potentially guiding prognosis and tailored therapeutic approaches.

**TABLE 6:** Relationship between all possible combined genotypes of *FCGR2A* and *FCGR3A* polymorphism and severity of the case.

*FCGR3A* 158F/V	*FCGR2A* 131H/R	Absolute/ 40	%	Acute	Chronic	Refractory	Persistent
FV	HR	13	32.5	23.07692	69.23077	0	7.6923077
FV	RR	4	10	100	0	0	0
FV	HH	5	12.5	20	60	0	20
VV	HR	5	12.5	80	20	0	0
VV	RR	1	2.5	100	0	0	0
VV	HH	2	5	0	50	50	0
FF	HR	7	17.5	14.3	57.1	14.3	14.3
FF	RR	3	7.5	33.3	66.7	0	0
FF	HH	0	0	0	0	0	0

## Discussion

This study investigated the association of *FCGR2A* (131H/R) and *FCGR3A* (158F/V) polymorphisms with disease susceptibility, chronicity, and severity in pediatric ITP, with a specific focus on gender-based genetic distributions. Consistent with prior research in pediatric populations, we did not observe statistically significant differences in overall genotype or allele frequencies between ITP patients and healthy controls, with *FCGR2A*-HR and *FCGR3A*-FV showing similar distributions across groups (10–12). However, exploratory analyses revealed that the HR/FV combined genotype was more prevalent among patients with chronic ITP, and certain sex-specific trends were observed, including higher frequencies of *FCGR2A*-HH and *FCGR3A*-VV in male patients. These observations suggest a potential influence of sex on genotype distribution, which may reflect the modulatory effects of sex hormones or sex-linked immune mechanisms on Fcγ receptor function. Nonetheless, these findings are based on small absolute numbers, particularly for rare genotypes such as VV/HH, and did not reach statistical significance, indicating that they should be interpreted as hypothesis-generating rather than confirmatory. The biological relevance of the identified variants remains uncertain, as functional assays assessing Fcγ receptor expression, IgG binding, or downstream effector functions were not performed. Furthermore, the PCR-RFLP methodology employed in this study detects only single nucleotide polymorphisms and cannot capture copy number variations, which are common in the FcγR gene cluster and have been shown to influence ITP susceptibility and therapeutic response. These methodological limitations reflect constraints in both budget and available laboratory techniques at our institution. Despite these limitations, the study provides preliminary insights into combined genotype associations and sex-specific patterns in a pediatric Middle Eastern cohort, a population that is underrepresented in current immunogenetic research. Our findings underscore the need for larger multicenter studies to validate these genotype-phenotype associations, functional analyses to establish biological significance, and incorporation of structural variant detection to fully characterize the FcγR locus. Moreover, understanding the impact of these variants on treatment response, including IVIg or thrombopoietin receptor agonists, may inform future personalized therapeutic strategies. In conclusion, while *FCGR2A* and *FCGR3A* polymorphisms alone were not significantly associated with ITP susceptibility, specific combined genotypes and gender-related trends appear to influence disease course and chronicity. These preliminary observations highlight the potential utility of immunogenetic profiling as a framework for hypothesis generation and for guiding future studies aimed at improving prognostic accuracy and individualized management in pediatric ITP.

## Data Availability

The datasets presented in this study can be found in online repositories. The names of the repository/repositories and accession number(s) can be found in the article/supplementary material.
